# SOX4 reprograms fatty acid metabolism through the CHREBP to inhibit ferroptosis in hepatocellular carcinoma

**DOI:** 10.1038/s41420-025-02527-4

**Published:** 2025-05-21

**Authors:** Fan Zhang, Zhiwei Wu, Yang Xiang, Qing He, Wanqing Li, Kaipeng Yang, Yijun Yang

**Affiliations:** 1 Department of Hepatobiliary Surgery, Affiliated Haikou Hospital of Xiangya Medical College, Central South University, Haikou, China; 2 Haikou Key Laboratory of Clinical Research and Transformation of Digestive Diseases, Haikou, China; 3 Department of Organ Transplantation, Xiangya Hospital, Central South University, Changsha, China; 4 Hunan Occupational Disease Prevention Hospital, Changsha, China

**Keywords:** Cancer metabolism, Oncogenes

## Abstract

Hepatocellular carcinoma (HCC) is a leading cause of cancer mortality, characterized by aggressive progression and poor prognosis. Pathological angiogenesis in HCC is closely linked to metabolic reprogramming, particularly concerning fatty acid metabolism. The interplay between fatty acid metabolism and ferroptosis, a type of cell death driven by lipid peroxidation, is emerging as a crucial area of study. The transcription factor SOX4 is known to be overexpressed in various cancers, including HCC, and may play a key role in these processes. We assessed SOX4 expression in HCC using clinical samples and data from online databases. Next-generation RNA sequencing was employed to explore the effects of SOX4 on fatty acid metabolism, focusing on the CHREBP pathway. Functional assays, including lipid peroxidation and angiogenesis studies, were conducted to investigate the role of SOX4 in regulating ferroptosis and angiogenesis in HCC. SOX4 was found to be significantly upregulated in HCC and associated with enhanced angiogenesis. Mechanistically, SOX4 activated the CHREBP/SCD1 pathway, leading to increased production of monounsaturated fatty acids, which in turn inhibited ferroptosis. This suppression of ferroptosis contributed to the promotion of angiogenesis and tumor progression in HCC. In conclusion, SOX4 reprograms fatty acid metabolism via the CHREBP/SCD1 pathway, thereby inhibiting ferroptosis and promoting angiogenesis in HCC. These findings suggest that targeting the SOX4-CHREBP axis could represent a novel therapeutic strategy for HCC.

## Introduction

Hepatocellular carcinoma (HCC) is one of the main cancer-related causes of death worldwide [1], accounts for about 90% of all primary liver cancers and is the second leading cause of cancer-related deaths worldwide [2]. Despite considerable advances in medical technology, the prognosis for HCC patients remains poor due to the aggressive nature of the disease, characterized by rapid growth, metastasis, and resistance to therapy. A significant challenge in HCC is its hypervascularity, which drives tumor growth and progression [3, 4]. Numerous approaches targeting angiogenesis have shown limited success in the clinical application of HCC [5, 6], Therefore, It remains crucial to investigate the underlying molecular mechanisms that drive abnormal vascularization in liver cancer.

Metabolic reprogramming is increasingly regarded as a core hallmark of cancer [7, 8]. Angiogenesis, another intricate hallmark crucial for cancer progress, has been reported the association with many metabolisms [9], including fatty acid metabolism [10–12]. Given that HCC is a highly metabolic cancer, its metabolism reprogramming has also been a focal point of research [13, 14]. Fatty acids not only fuel the energy demands of cancer cells but also play a crucial role in modulating angiogenesis through the secretion of pro-angiogenic factors [15]. Targeting fatty acid metabolism in cancer cells has emerged as a potential anti-cancer strategy.

In addition to its role in supporting angiogenesis, fatty acid metabolism is also tightly linked to ferroptosis—a regulated form of cell death driven by the accumulation of lipid peroxides. Polyunsaturated fatty acids (PUFAs) within cellular membranes are particularly susceptible to peroxidation, leading to ferroptosis [16]. In contrast, The generation of monounsaturated fatty acids (MUFAs) inhibits this process [17]. Therefore, it is reasonable to believe that certain pathogenic factors may regulate ferroptosis through fatty acid metabolism, thereby further promoting angiogenesis in hepatocellular carcinoma. However, the mechanistic links between these processes remain poorly understood.

SOX4, an integral member of the SOX (SRY-related HMG box) family, has garnered crucial attention for its pivotal role in the pathogenesis of HCC. Elevated expression of SOX4 has been associated with poor prognosis in several cancers, including colorectal [18], lung [19], breast [20], and notably, HCC. More recently, SOX4 has been recognized as a novel pro-angiogenic regulator, modulate the expression of endothelin-1 (ET-1) and thereby promoting tumor-induced angiogenesis [21]. It also activates CXCL12 transcriptionally, thereby fostering angiogenesis in HCC [22]. Unfortunately, there is currently a lack of research exploring the interplay between SOX4, ferroptosis and fatty acid metabolic reprogramming in HCC. Understanding how SOX4 influences these processes could uncover new therapeutic opportunities for targeting the metabolic vulnerabilities and vascularization of HCC.

In this study, we, for the first time, linked SOX4’s potential to regulate fatty acid metabolism and its ferroptosis-inhibitory effects to angiogenesis in HCC. We validated the expression level of SOX4 in HCC using clinical samples and online data. And analyzed its regulatory capacity in angiogenesis. Utilizing clinical sample-derived next-generation RNA-seq, we discern its regulatory mechanism, focusing on the fatty acid metabolism. And further study proved that SOX4 may regulate ferroptosis through reprograming fatty acid metabolism in HCC. To be more specific, SOX4 activates the CHREBP signaling pathway, leading to the upregulation of multiple proteins involved in fatty acid synthesis. Collectively, our findings uncover a previously unrecognized axis through which SOX4 regulates fatty acid metabolism and ferroptosis to promote angiogenesis, offering potential therapeutic targets for HCC.

## Results

### SOX4 is highly expressed in HCC and is closely associated with angiogenesis

**Fig. 1:**
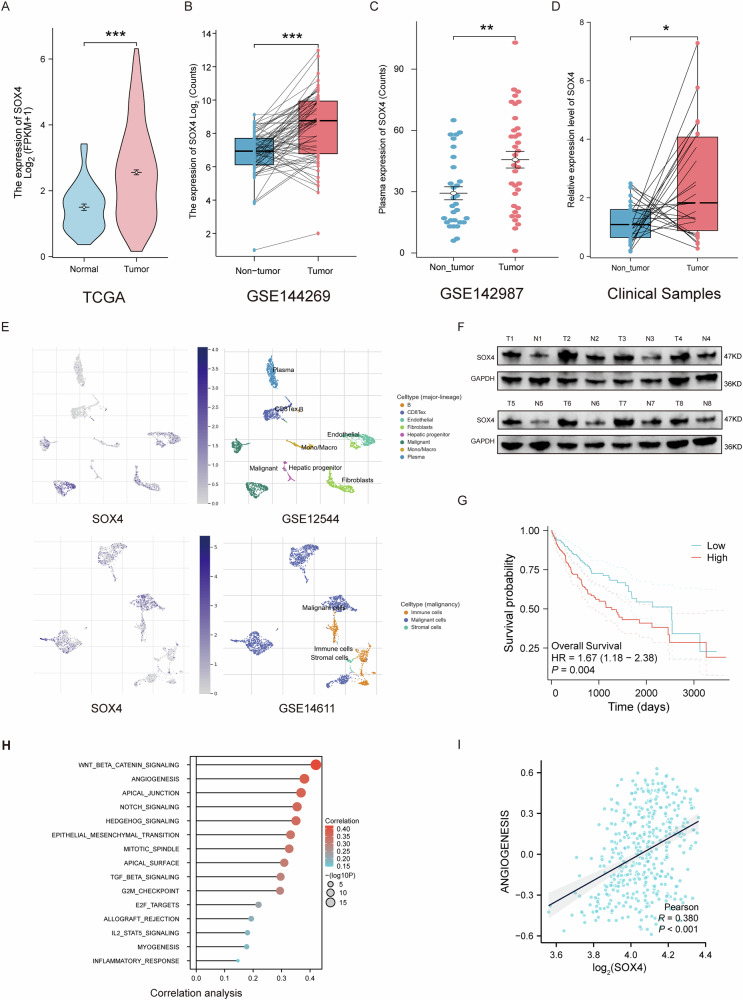
SOX4 is overexpressed in HCC and correlates with angiogenesis. **A**, **B** SOX4 mRNA levels are significantly higher in HCC tumor tissues compared to normal tissues based on TCGA and GSE144269 datasets. **C** Elevated SOX4 levels were detected in the plasma of HCC patients using GSE142987. **D** Analysis of paired tumor and adjacent non-tumor tissues confirmed high SOX4 expression in tumor tissues using clinical samples. **E** Single-cell transcriptomics analysis (GSE12544, GSE14611) shows predominant SOX4 expression in malignant cells. **F** Western blot analysis validates elevated SOX4 protein levels in tumor tissues. **G** Kaplan–Meier survival curves from TCGA demonstrate poorer survival in patients with high SOX4 expression. **H**, **I** Gene Set Variation Analysis (GSVA) reveals a significant association between SOX4 and the angiogenesis hallmark. *P < 0.05, **P < 0.01, ***P < 0.001.

To investigate the expression levels of SOX4 in hepatocellular carcinoma (HCC), we first analyzed its mRNA expression using the TCGA database and dataset GSE144269. The results revealed that SOX4 is significantly overexpressed in tumor tissues (Fig. 1A, B). Notably, SOX4 levels were also markedly elevated in the plasma of HCC patients, suggesting its potential as a non-invasive diagnostic biomarker for HCC (Fig. 1C). We further validated these findings by analyzing paired HCC and adjacent non-tumor tissues, confirming that SOX4 is predominantly expressed in HCC tissues (Fig. 1D). At the single-cell level, analyses of datasets GSE12544 and GSE14611 demonstrated that SOX4 is mainly expressed in Malignant cells, with partial expression in epithelial cells (Fig. 1E). Protein-level analysis also confirmed a similar trend of elevated SOX4 expression in tumor tissues (Fig. 1F). Furthermore, survival analysis based on the TCGA database indicated that high SOX4 expression is associated with significantly poorer prognosis (Fig. 1G). Gene Set Variation Analysis (GSVA) revealed a significant correlation between SOX4 expression and the angiogenesis hallmark, suggesting that aberrant angiogenesis is a key pathological process influenced by SOX4 (Fig. 1H, I and Supplementary Table 4).

### In vitro and in vivo assays demonstrate the oncogenic role of SOX4 in HCC

**Fig. 2:**
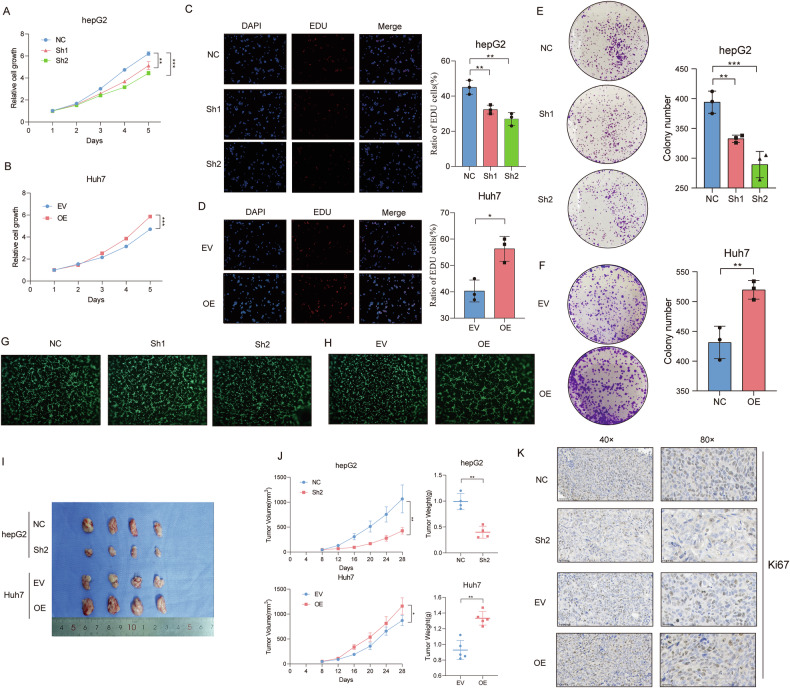
SOX4 promotes proliferation, colony formation, and angiogenesis in vitro and tumor growth in vivo. **A**, **B** CCK-8 assays show that SOX4 knockdown (HepG2 cells) reduces cell proliferation, while overexpression (Huh7 cells) enhances proliferation. **C**, **D** EDU assays confirm decreased proliferation rates upon SOX4 knockdown and increased rates with SOX4 overexpression. **E**, **F** Colony formation assays show reduced colony formation in SOX4 knockdown cells and enhanced formation in overexpressing cells. **G**, **H** Fluorescence-labeled angiogenesis assays reveal SOX4’s role in promoting angiogenesis in vitro. **I**, **J** Xenograft experiments show reduced tumor growth and volume in SOX4 knockdown cells and increased tumor growth in overexpressing cells. **K** Immunohistochemistry further validates SOX4 protein levels in tumor tissues.*P < 0.05, **P < 0.01, ***P < 0.001.

To further explore the role of SOX4, we established stable SOX4 knockdown and overexpression cell lines. SOX4 shRNA in HepG2 cells exhibited good knockdown efficiency, with sh2 showing the best knockdown effect. We used sh1 and sh2 in the subsequent experiments (Fig. S1A, B). Stable SOX4 overexpression in Huh7 cells was also validated (Figures S1C-S1D). CCK-8 assays revealed that SOX4 knockdown significantly reduced the growth rate of HCC cells (Fig. 2A), whereas SOX4 overexpression promoted their growth (Fig. 2B). EdU assays revealed that SOX4 knockdown significantly decreased the proliferation rate of HCC cells **(**Fig. 2C), whereas SOX4 overexpression markedly enhanced their proliferation **(**Fig. 2D). In the colony formation assays, we observed that SOX4 knockdown led to a suppression of the number of colonies in HepG2 cells **(**Fig. 2E), while SOX4 overexpression significantly increased colony formation in Huh7 cells **(**Fig. 2F). To investigate the relationship between SOX4 and angiogenesis, we performed fluorescence-labeled angiogenesis assay using HUVEC cells. The results demonstrated that SOX4 significantly influences angiogenesis in vitro (Fig. 2G, H), consistent with our previous analysis. We selected the sh2 construct, which demonstrated the best performance in vitro, for subsequent in vivo experiments. In vivo validation using a xenograft tumor formation assay (Fig. 2I) showed that SOX4 knockdown in the HepG2 cell line significantly reduced tumor growth rate and final tumor volume Conversely, SOX4 overexpression in Huh7 cells accelerated tumor growth and increased final tumor volume (Fig. 2J). In addition, the expression level of SOX4 in tumors was further validated through immunohistochemistry (Fig. 2K).

### SOX4 drives HCC progression by reprogramming fatty acid metabolism

**Fig. 3:**
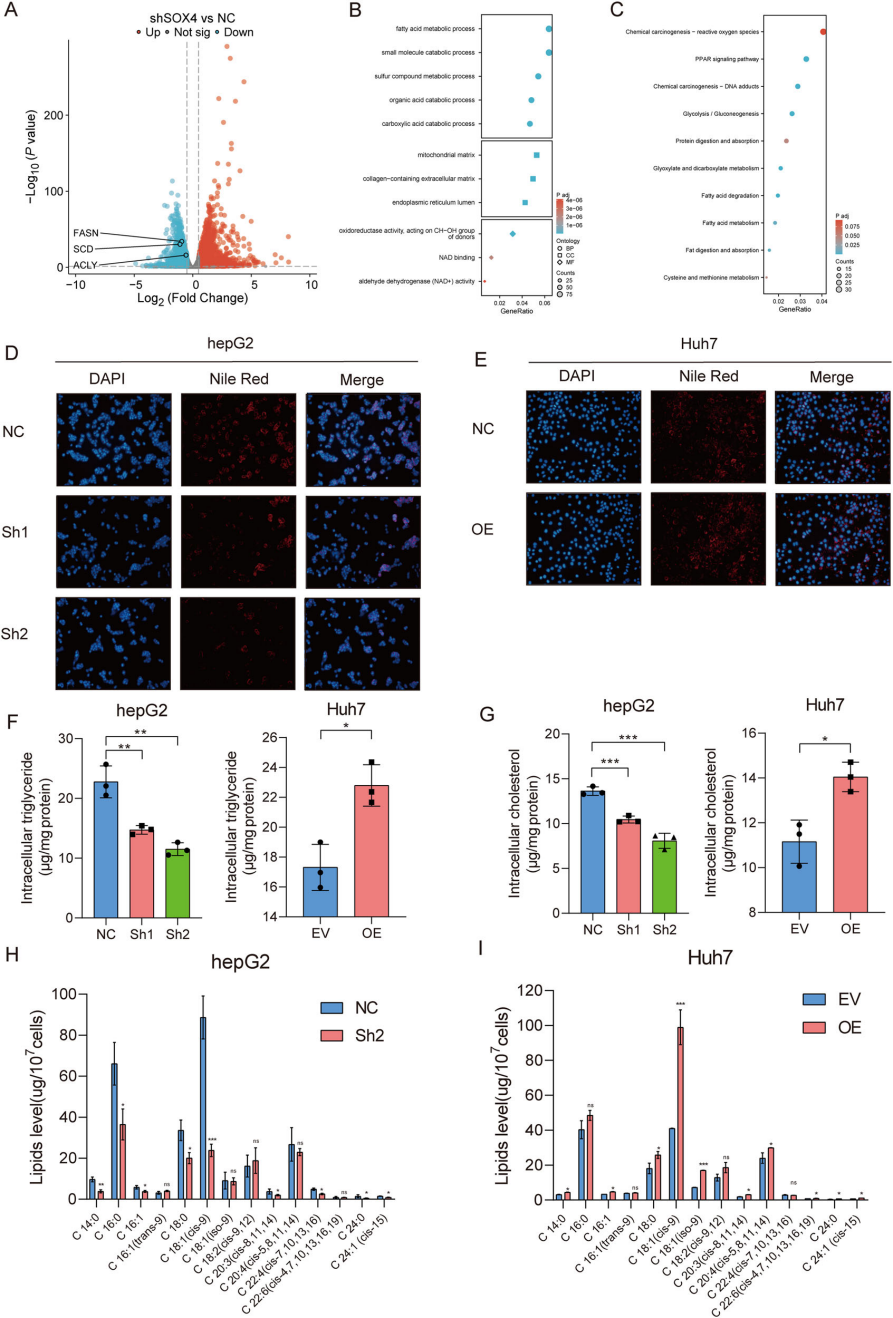
SOX4 drives fatty acid metabolism reprogramming in HCC. **A** RNA-sequencing identifies differentially expressed genes upon SOX4 knockdown (2248 upregulated and 1748 downregulated). **B**, **C** GO and KEGG analyses highlight significant enrichment in fatty acid metabolism pathways. **D**, **E** Nile Red staining shows decreased lipid droplet accumulation in SOX4 knockdown cells and increased accumulation in overexpressing cells. **F**, **G** Intracellular triglyceride and cholesterol levels are significantly altered by SOX4 modulation. **H**, **I** GC–MS analysis confirms reduced fatty acid levels in SOX4 knockdown cells and increased levels in overexpressing cells. *P < 0.05, **P < 0.01, ***P < 0.001.

To elucidate the mechanisms by which SOX4 exerts its effects, we performed RNA sequencing to identify genes potentially regulated by SOX4. Differential expression analysis revealed that 2248 genes were upregulated, while 1748 genes were downregulated (Figs. 3A and S2A, B). GO analysis of the downregulated genes showed significant enrichment in fatty acid metabolism (Fig. 3B), suggesting that SOX4 may promote HCC progression via fatty acid metabolism reprogramming. KEGG analysis also found that fatty acid metabolism and fatty acid degradation pathways were significantly enriched (Fig. 3C). Protein-protein interactions among differentially expressed genes regulated by SOX4 revealed that FABP1 and other fatty acid metabolism-related genes occupy central positions in the network (Fig. S2C). Nile red staining showed that lipid levels were significantly decreased in HCC cells with SOX4 knockdown (Fig. 3D), whereas SOX4 overexpression led to a notable increase in lipid levels (Fig. 3E). The regulation of SOX4 significantly altered the expression levels of triglycerides in liver cancer cells (Fig. 3F) and cholesterol (Fig. 3G). GC-MS analysis further confirmed that SOX4 knockdown led to reduced expression levels of various fatty acids (Fig. 3H), while SOX4 overexpression resulted in the accumulation of many fatty acids (Fig. 3I**)**. CCK-8 and ROS assays revealed that exogenous supplementation of 1 μM C18:1 (cis-9) partially rescued the ferroptosis-promoting effects induced by SOX4 knockdown (Fig. S3A, B). More specifically, our study demonstrates that SOX4 markedly enhances the accumulation of monounsaturated fatty acid(MUFAs) oleic acid (C18:1) and saturated fatty acid palmitic acid (C16:0) in HCC cells, which effectively suppress ferroptosis through their antioxidant properties. Overall, these findings suggest that SOX4 may contribute to lipid accumulation by modulating fatty acid metabolism.

### SOX4 inhibits ferroptosis by modulating fatty acid metabolism in HCC

**Fig. 4:**
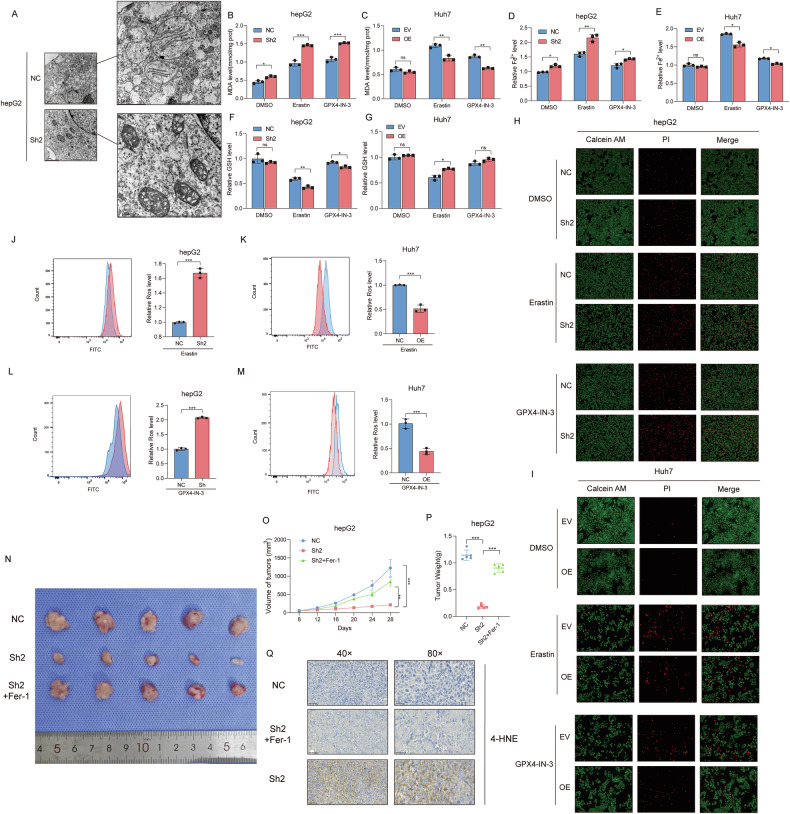
SOX4 inhibits ferroptosis by modulating fatty acid metabolism. **A** Transmission electron microscopy shows mitochondrial shrinkage, a hallmark of ferroptosis, in SOX4 knockdown cells. **B**, **C** MDA levels are increased in knockdown cells and decreased in overexpressing cells upon erastin treatment. **D**, **E** SOX4 regulates Fe²⁺ levels during ferroptosis after incubation of Erastin or GPX4-IN-3. **F**, **G** GSH levels are reduced in knockdown cells and increased in overexpressing cells during ferroptosis after incubation of Erastin or GPX4-IN-3. **H**, **I** Calcein AM/PI staining confirms ferroptosis-induced cell death is modulated by SOX4. **J**–**M** ROS accumulation is enhanced in SOX4 knockdown cells and suppressed in overexpressing cells. **N**–**P** Xenograft tumors treated with ferroptosis inhibitors show partial reversal of growth inhibition. **Q** Immunohistochemistry for 4-HNE in tumors indicates enhanced lipid peroxidation in SOX4-deficient tumors. ns *P* > 0.05, *P < 0.05, **P < 0.01, ***P < 0.001.

Fatty acid metabolism plays a critical role in ferroptosis by modulating lipid peroxidation, therefore, given the connection between fatty acid metabolism and ferroptosis, we further investigated whether SOX4 might regulate ferroptosis. Electron microscopy analysis demonstrated that SOX4 knockdown induced mitochondrial shrinkage, a hallmark of ferroptosis, indicating the activation of ferroptosis (Fig. 4A). MDA, a metabolic product commonly associated with lipid peroxidation during ferroptosis, was measured. The results showed that SOX4 knockdown promoted MDA production after ferroptosis was activated by erastin, while SOX4 overexpression significantly reduced MDA levels (Fig. 4B, C). Iron ion (Fe²⁺) levels, a critical mediator of lipid peroxidation during ferroptosis, were quantified using Ferrozine-based colorimetric assay. Consistent with MDA results, SOX4 knockdown significantly increased intracellular Fe²⁺ accumulation after erastin treatment (Fig. 4D), whereas SOX4 overexpression suppressed Fe²^+^levels (Fig. 4E). GSH levels in HCC cells were also as1sessed with erastin, revealing that SOX4 knockdown decreased GSH expression in the process of ferroptosis (Fig. 4F), whereas SOX4 overexpression led to an increase (Fig. 4G). Subsequently, we employed Calcein-AM/PI cell staining to evaluate the impact of SOX4 modulation on cell viability under ferroptosis-inducing conditions using two distinct ferroptosis activators (Erastin and GPX4-IN-3). The results revealed that SOX4 knockdown significantly exacerbated cell death induced by ferroptosis activators (Fig. 4H), whereas SOX4 overexpression attenuated the ferroptosis-promoting effects of these compounds (Fig. 4I). ROS accumulation, a hallmark of ferroptosis, was examined, and our findings indicated that SOX4 knockdown enhanced ROS production, suggesting inhibition of ferroptosis, while SOX4 overexpression suppressed ROS accumulation during this process (Fig. 4J, K). And subcutaneous xenograft tumors in nude mice showed that SOX4 knockdown potently inhibited tumor growth, reducing final volume by 60% compared with the control group. Notably, treatment with ferroptosis inhibitors Ferrostatin-1 partially reversed the growth-inhibitory effects (Fig. 4N–P). Immunohistochemical staining with an anti-4-HNE antibody demonstrated significantly elevated 4-HNE levels in SOX4-deficient tumors, indicating enhanced lipid peroxidation and oxidative stress (Fig. 4Q). These results indicate that SOX4 may inhibit ferroptosis in vitro and in vivo.

### SOX4 drives fatty acid metabolism reprogramming through transcriptional activation of ChREBP in HCC

**Fig. 5:**
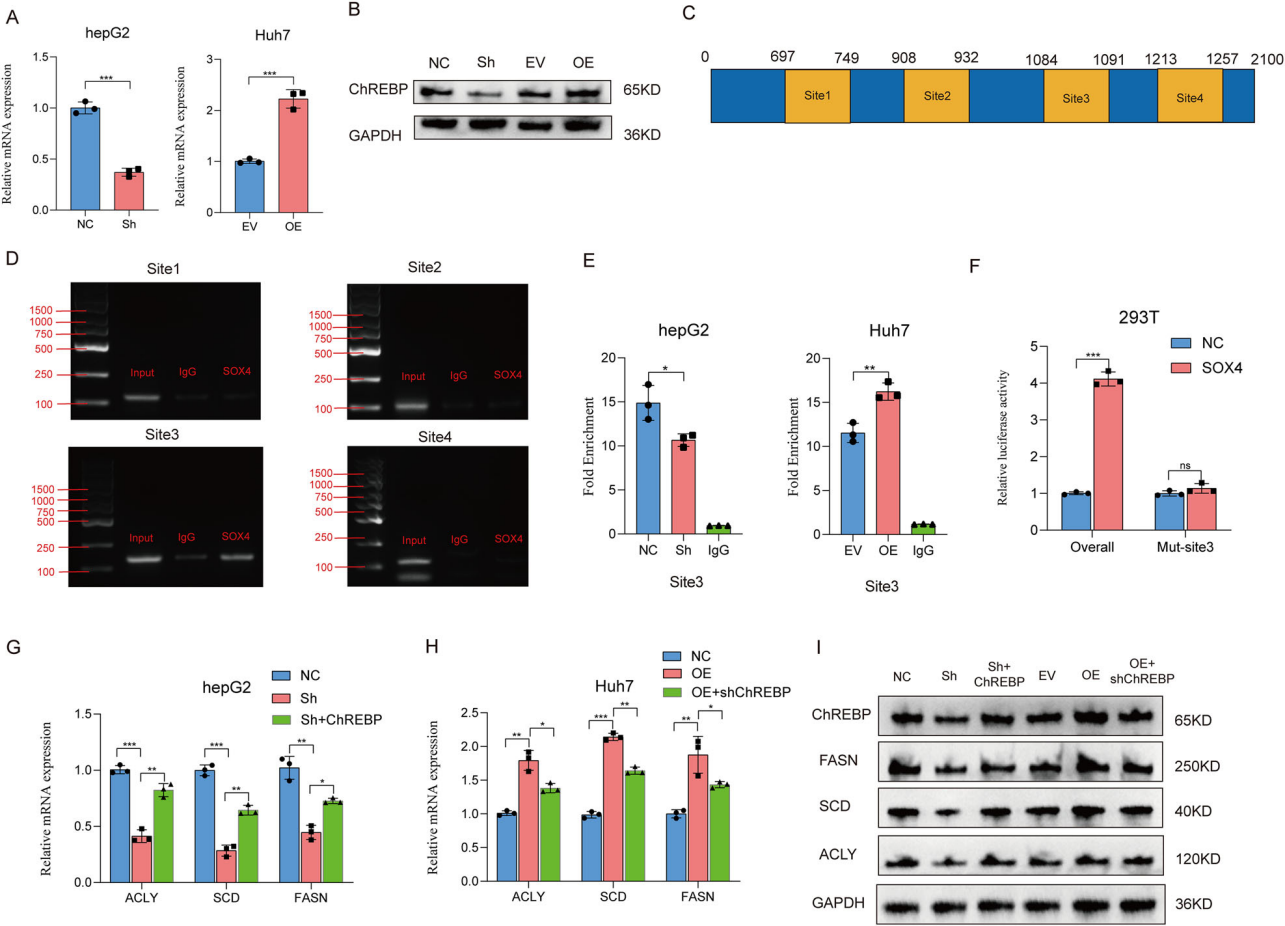
SOX4 directly regulates ChREBP to modulate fatty acid metabolism in HCC. **A**, **B** RT-qPCR and Western blot confirm SOX4’s regulation of ChREBP mRNA and protein levels. **C** JASPAR analysis identifies binding sites in the ChREBP promoter region. **D**, **E** ChIP assay shows SOX4 binds to the ChREBP promoter at site 3, which is enhanced upon SOX4 overexpression. **F** Dual-luciferase assays confirm the specific binding of SOX4 to ChREBP, and notably, mutation of the binding site (site 3) results in a significant reduction in fluorescence intensity. **G**, **H** RT-qPCR demonstrates that ChREBP overexpression rescues the effects of SOX4 knockdown on fatty acid metabolism-related genes (ACLY, SCD, FASN), while ChREBP knockdown reverses the effects of SOX4 overexpression. **I** Western blot analysis shows that overexpression of ChREBP rescues the effects of SOX4 knockdown on fatty acid metabolism-related proteins (ACLY, SCD, FASN), while knockdown of ChREBP reverses the effects of SOX4 overexpression. *P < 0.05, **P < 0.01, ***P < 0.001.

Since previous studies have shown that MLXIPL (ChREBP) broadly regulates the reprogram of fatty metabolism, we hypothesized that SOX4 might regulate these downstream proteins through ChREBP. NGS data revealed that SOX4 knockdown significantly altered the expression levels of MLXIPL (Fig. S4A), RT-qPCR suggested that SOX4 indeed influences ChREBP mRNA levels (Fig. 5A) a finding further supported by WB analysis, which demonstrated changes in ChREBP protein levels following SOX4 modulation (Fig. 5B). As a transcription factor, SOX4 is known to regulate downstream gene transcription. Using the JASPAR database, we identified multiple high-scoring potential binding sites for SOX4 in the ChREBP promoter region (Fig. 5C). The gel electrophoresis results from the CHIP assay revealed that site3 serves as the binding site for both SOX4 and ChREBP. (Fig. 5D). ChIP-qPCR assays revealed that SOX4 knockdown weakened the binding at site 3, whereas SOX4 overexpression significantly enhanced the binding at site 3 (Fig. 5E), further confirming that the binding site between SOX4 and ChREBP is located at site 3. In a dual-luciferase reporter assay, the wild-type ChREBP probe resulted in a significant increase in luciferase activity, indicating strong binding to the target sequence. Conversely, the mut-site3 ChREBP probe, which harbors a specific mutation in the binding site, showed a marked reduction in luciferase activity, confirming the specificity of the interaction (Fig. 5F). NGS data revealed that SOX4 knockdown significantly altered the expression levels of fatty acid metabolism proteins, including ACLY, SCD, and FASN (Fig. S4B). Importantly, RT-qPCR demonstrated that overexpression of ChREBP in SOX4-knockdown cells significantly rescued the inhibition of fatty acid metabolism-related genes, including ACLY, SCD, and FASN, caused by SOX4 knockdown in HCC cells (Fig. 5G). Conversely, knockdown of ChREBP in SOX4-overexpressing cells showed the opposite trend (Fig. 5H). Western blotting also supported the regulatory effect of SOX4 (Fig. 5I).

### SOX4/ChREBP Axis Regulates Lipid Metabolism and Ferroptosis in HCC

**Fig. 6:**
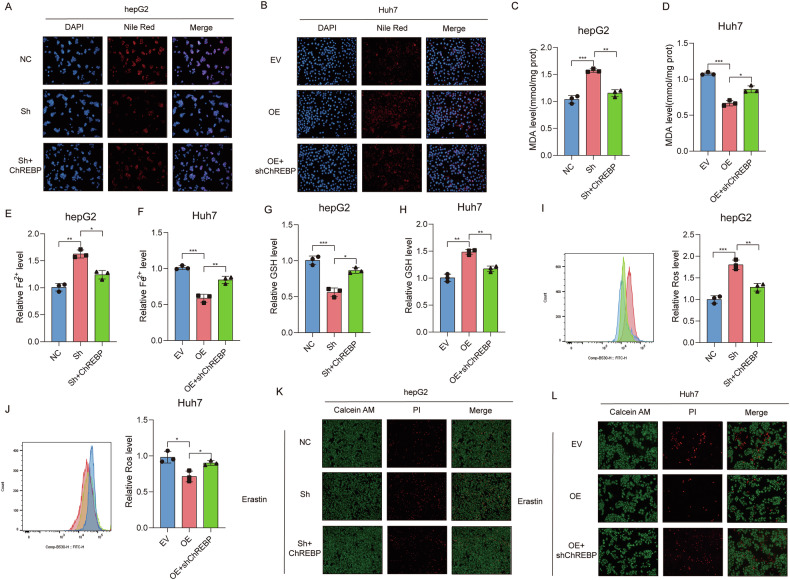
The SOX4/ChREBP axis regulates lipid metabolism and ferroptosis. **A**, **B** Nile Red staining demonstrates SOX4 knockdown reduces lipid accumulation, rescued by ChREBP overexpression, while SOX4 overexpression increases lipid droplets, reversed by ChREBP knockdown. **C**, **D** MDA levels during ferroptosis are modulated via the SOX4/ChREBP axis. **E**, **F** Fe²⁺ levels during ferroptosis are regulated by the SOX4/ChREBP axis. **G**, **H** GSH levels are significantly altered during ferroptosis through the SOX4/ChREBP axis. **I**, **J** ROS production is regulated by the axis, as shown by flow cytometry. **K**, **L** Calcein AM/PI staining highlights the effect of the SOX4/ChREBP axis on ferroptosis-induced cell death. *P < 0.05, **P < 0.01, **P < 0.001.

To investigate whether SOX4 promotes hepatocellular carcinoma (HCC) progression via ChREBP, we conducted a series of functional rescue assays. First, we validated the regulatory efficiency of the ChREBP overexpression plasmid and the shRNA of ChREBP (Fig. S5A–D), and we selected the sh3 with the best knockdown efficiency for further validation. Nile Red staining demonstrated that knockdown of SOX4 in HepG2 cells significantly reduced lipid droplet accumulation. However, this reduction was partially restored by overexpression of ChREBP (Fig. 6A). In contrast, SOX4 overexpression combined with ChREBP knockdown induced a marked increase in lipid droplet accumulation (Fig. 6B). Subsequently, we examined whether the inhibition of ferroptosis by SOX4 occurs through the SOX4/ChREBP axis. Measurements of MDA, Fe²⁺, and GSH levels in cancer cells revealed that SOX4 knockdown enhanced ferroptosis, while ChREBP overexpression partially reversed this enhancement (Fig. 6C, E, G). Conversely, the combination of SOX4 overexpression and ChREBP knockdown resulted in a pronounced suppression of ferroptosis (Fig. 6D, F, H). Furthermore, flow cytometry analysis of ROS production indicated that the SOX4/ChREBP axis significantly modulates ROS levels during ferroptosis (Fig. 6I, J). Consistently, Calcein AM/PI double staining confirmed that manipulation of the SOX4/ChREBP axis alters the number of dying cells (Fig. 6K, L).

## Conclusion

In conclusion, our in vitro and in vivo studies demonstrate that SOX4 regulates Fatty acid metabolism through ChREBP, thereby inhibiting ferroptosis and promoting angiogenesis and tumor growth in HCC.

## Discussion

In this study, using the bioinformatic analysis as previous study of ours [23], we found that SOX4 is highly correlated with the process of Angiogenesis. Then, we identified SOX4 as a potential angiogenesis-promoting factor that is strongly correlated with the reprogramming of fatty acid metabolism. This is the result on which no prior study has ever conducted. In previous studies, SOX4 has been repeatedly demonstrated to promote the progression of HCC. For instance, Chi-Neu Tsai et al. discovered that SOX4 facilitates angiogenesis in HCC by activating the CXCL12 signaling pathway [22]. Similarly, Wonhee Hur et al. found that SOX4 promotes HCC development by inhibiting p53-mediated apoptosis [24]. Other studies have also shown the significant connection between SOX4 and immune cell infiltration in HCC. For example, A study suggested that SOX4 may influence HCC progression by regulating lymphocyte differentiation [25]. Due to its strong pro-tumorigenic effects in HCC, numerous studies have explored the upstream regulatory genes of SOX4, identifying it as a specific target for non-coding RNAs that drive HCC progression [26, 27]. However, previous research has never explored the metabolic reprogramming genes regulated by SOX4. Considering that SOX4 is a transcription factor capable of modulating the transcription of many downstream genes, its role in regulating metabolic reprogramming targets should not be neglected. In this study, using the NGS, we observed that knockdown of SOX4 led to significant changes in the expression levels of various genes related to fatty acid metabolism, including ACLY, SCD1, and FASN. Based on previous studies, we identified MLXIPL, also known as ChREBP, as a common regulatory factor for these fatty acid metabolism genes [28]. As a highly metabolic cancer, the progression of HCC depends on aberrant lipogenesis induced by various factors, making the role of ChREBP particularly critical [29]. It functions by binding to carbohydrate response elements in the promoters of target genes involved in regulating glucose and lipid metabolism. A recent study revealed that ChREBP reroutes glucose and glutamine metabolic fluxes into fatty acid and nucleic acid synthesis to support the progress of HCC [30]. However, However, the role of ChREBP in cancer remains controversial, likely due to context-dependent effects across tumor types. In gastric cancer, for example, ChREBP has been found to exert tumor-suppressive effects. This may be influenced by the distinct metabolic contexts of different cancer types [31]. In this study, we found that modulating SOX4 significantly changed ChREBP mRNA expression, and further experiments confirmed that SOX4 functions as a transcriptional regulator of ChREBP. More importantly, through ChIP-qPCR, for the first time, we validated that SOX4 effectively binds to the specific site on the ChREBP promoter. In summary, through SOX4/ChREBP axis, we effectively explained the reason for SOX4 altering the levels of various fatty acid metabolism genes in HCC, thereby regulating fatty acid metabolism reprogramming. This discovery addresses a significant gap in the current understanding of SOX4’s role in the metabolic alterations associated with HCC.

Meanwhile, SCD1 is a significantly upregulated fatty acid metabolism molecule induced by SOX4. SCD1 plays a critical role in inhibiting ferroptosis in cancers, Mechanistically, SCD1 decreases the availability of oxidizable lipids, thereby inhibiting ferroptosis [16, 32, 33]. There are no reports indicating that ACLY and FASN can directly regulate ferroptosis. However, they both are closely connected to SCD1 in HCC [34]. We also investigated the SOX4/ChREBP axis on ferroptosis. Our results revealed that SOX4 significantly inhibits ferroptosis.

Notably, we demonstrated that SOX4 upregulation leads to the reprogramming of fatty acid metabolism, resulting in aberrant lipid accumulation that inhibits ferroptosis. This initially appears to contradict existing research, which typically suggests that abnormal fatty acid accumulation induces ferroptosis through lipid peroxidation [35, 36]. However, our findings reveal that the lipid accumulation driven by SOX4 is primarily composed of MUFAs and saturated fatty acid, which are known to inhibit ferroptosis [37, 38]. Previous studies have also shown that the biological function of SCD1 is to cause the abnormal accumulation of MUFAs [39, 40]. Therefore, we can reasonable conjectured that although SOX4 leads to the accumulation of certain fatty acids, it predominantly facilitates the accumulation of MUFAs. Consequently, SOX4 inhibits tumor ferroptosis through the reprogramming of fatty acid metabolism.

Collectively, our findings established a previously unrecognized SOX4/ChREBP axis that promotes HCC progression by reprogramming fatty acid metabolism and inhibiting ferroptosis. This finding provides a new perspective for targeted therapy in HCC.

## Methods

### Cell lines and culture conditions

Cell lines were cultured as previous described [41]. Briefly, to establish and maintain the breast cancer cell lines, including HepG2(ATCC HB-8065) and Huh7 (JCRB 0403) cells. All cell lines have undergone STR profiling and have been confirmed to be free of mycoplasma contamination.The cells were cultured under controlled conditions in a CO_2_ incubator. Cells were cultured in DMEM (Dulbecco’s Modified Eagle Medium) supplemented with 10% fetal bovine serum (FBS) and 1% penicillin/streptomycin at 37 °C with 5% CO₂. The media were replaced every 3 days using 0.25% trypsin-EDTA at a 1:4 split ratio to ensure optimal growth conditions and nutrient availability, and routinely tested for mycoplasma contamination using PlasmoTest (InvivoGen).

### Plasmid and lentivirus

Plasmids designed for gene overexpression and interference were purchased from Tsingke Biotech. SOX4 Knockdown: HepG2 cells were transfected with SOX4-specific shRNA lentivirus (shSOX4) using Lipofectamine 3000 (Invitrogen). Stable knockdown cells were selected using 2 μg/mL puromycin. SOX4 Overexpression: Huh7 cells were transfected with SOX4 overexpression plasmid (pCDH-SOX4) using Lipofectamine 3000. Stable overexpression cells were selected with 500 μg/mL G418.

### qRT-PCR analysis

Total RNA was extracted using TRIzol reagent (Invitrogen). cDNA synthesis was carried out, which facilitates efficient conversion of RNA into complementary DNA. For the qRT-PCR, SYBR Green SuperMix (Yeasen) was utilized, providing a highly sensitive and specific method for quantifying gene expression. ΔΔCt method normalized to GAPDH. The qRT - PCR analysis was repeated 3 times for each sample. Detailed information on the primers used is available in Supplementary Table 1.

### Western blotting

Proteins were extracted using the RIPA Lysis Buffer from Vazyme Biotech. Proteins (30 μg/lane) were separated on 10% SDS-PAGE at 80 V for 30 min (stacking gel) and 120 V for 60 min (separating gel) for electrophoresis. Following electrophoresis, proteins were transferred onto PVDF membranes (Millipore) to facilitate antibody-based detection. The membranes were blocked using 5% BSA. Primary antibodies were incubated with the protein overnight at 4 °C to specifically bind to the target proteins. After washing with Tris Buffered Saline with Tween (TBST), secondary antibodies conjugated with HRP (horseradish peroxidase) were added for 1 hour. The details of antibodies used are provided in Supplementary Table 2.

### In vitro functional assays

#### CCK-8 assays

Cell proliferation was assessed using CCK-8 assays (Dojindo). 5 × 10^3^ cells/well were seeded in 96-well plates and incubated for 24, 48, and 72 h. Absorbance at 450 nm was measured.This assay was repeated 3 times independently.

#### Colony formation assays

Five hundred cells per well were plated in 6-well plates and allowed to form colonies for 2 weeks. Colonies were fixed with methanol (10 min) and stained with 0.1% crystal violet for 30 min. Colonies were counted under a microscope. The colony formation assay was repeated 3 times.

### Angiogenesis assays

HUVEC cells were seeded in 96-well plates coated with Matrigel (Corning,356234). SOX4 knockdown and overexpression cell-conditioned media were used to assess tube formation. Images were captured using a microscope, and tube formation was quantified using ImageJ software.

### RNA sequencing and analysis

RNA was isolated from SOX4 knockdown and control cells (*n* = 3/group). Libraries were prepared and sequenced by Aifang Int. Differential gene expression analysis was performed using DESeq2 package in R (|log2FC| > 1, FDR < 0.05).

Enrichment analysis was conducted using the clusterProfiler package in R.

### Fatty acid metabolism analysis

#### Lipid staining

Nile red staining was performed to visualize lipid levels. Cells were stained with Nile red solution (1 μg/mL) and imaged using a fluorescence microscope.

#### GC-MS analysis

Fatty acid composition was analyzed using Gas Chromatography-Mass Spectrometry (GC-MS). Lipids were extracted from cell samples and analyzed according to standard protocols. *n* = 3 biological replicates per group. All drugs/reagents were listed in Supplementary Table 3.

### Ferroptosis assays

The ferroptosis assays were conducted followed by our previous study [41], MDA Assay: Malondialdehyde (MDA) levels were quantified using an MDA assay kit (Abcam, ab118970) with 100 μL cell lysate per well, incubated at 95 °C for 60 min. Absorbance was measured at 532 nm using a microplate reader. or GSH detection, cells (1 × 10⁶) were lysed in 100 μL ice-cold buffer, and 50 μL supernatant was reacted with 5,5’-dithiobis(2-nitrobenzoic acid) (DTNB) reagent (BioVision, K264-100). Fluorescence intensity (Ex/Em = 405/480 nm) was recorded using a microplate reader.

### In vivo experiments

All animal procedures in this in vivo study were conducted in strict accordance with the guidelines of the Xiangya Hospital Laboratory Animal Ethics Committee (Approval No. XY20240913005).6-week-old male BALB/c nude mice (*n* = 5/group, purchased from Hunan SJA Laboratory Animal Co., Ltd. (Changsha, China)) were housed in a specific pathogen-free (SPF) facility with a 12-h light/dark cycle, a temperature of 22 ± 2 °C, and a humidity of 50 ± 10%, and provided with autoclaved food and water ad libitum. A random number generation tool was used to generate a random number for each animal, and the animal IDs were sorted based on these random numbers. According to the number of experimental groups N, the animals were divided into N groups in descending order of their sorted IDs. The trial was not blinded.SOX4 knockdown or control HCC cells (3 × 10⁶ cells) were subcutaneously injected into the right flank of each mouse. Tumor growth was monitored weekly, with tumor volumes measured using digital calipers and calculated by the formula Volume = (length × width²)/2. Mice were euthanized after 4 weeks, after which tumors were excised, weighed, and fixed in 4% paraformaldehyde for histological analysis. all statistical analyses were performed using GraphPad Prism 9.0 software.

### Chromatin immunoprecipitation (ChIP)

Chromatin immunoprecipitation (ChIP) assays were conducted using the ChIP kit (Millipore) to examine SOX4 binding to the ChREBP promoter. Cells were first crosslinked with 1% formaldehyde, and chromatin was then fragmented by sonication to obtain DNA fragments of 200–500 bp. Immunoprecipitation was performed with anti-SOX4 antibodies, and non-specific IgG was used as a negative control. The protein-DNA complexes were captured using magnetic beads, and following multiple washes, the bound DNA was eluted and reverse crosslinked. Purified DNA was analyzed by qPCR to quantify SOX4 binding at specific sites within the ChREBP promoter, with enrichment calculated relative to the input and IgG controls.

### Gene set variation analysis (GSVA)

Gene Set Variation Analysis (GSVA) was performed to evaluate the pathway activity of hallmark gene sets in hepatocellular carcinoma (LIHC) samples from The Cancer Genome Atlas (TCGA-LIHC) database. Normalized gene expression data were used as input for the GSVA algorithm, which assigns an enrichment score to each sample, reflecting the relative activity of each pathway in an unsupervised manner. The enrichment scores were subsequently analyzed using Spearman’s rank correlation to assess their association with SOX4 expression levels. All analyses were conducted using the GSVA R package.

### Dual-luciferase reporter assay

The dual-luciferase reporter assay was performed to evaluate the binding of SOX4 to the ChREBP promoter. Wild-type (WT) and mutant (Mut) ChREBP promoter sequences were cloned into a luciferase reporter vector (e.g., pGL3-basic). HepG2 and Huh7 cells were co-transfected with the luciferase constructs and a Renilla luciferase plasmid as a normalization control. After 48 hours of transfection, luciferase activities were measured using the Dual-Luciferase Reporter Assay System (Promega). Firefly luciferase activity was normalized to Renilla luciferase activity, and the relative luciferase activity was compared between WT and Mut promoters in SOX4-overexpressing and control cells. Increased luciferase activity in the WT promoter confirmed the transcriptional activation of ChREBP by SOX4, while decreased activity in the Mut promoter validated the specificity of the binding site.

### Statistical analysis

Statistical analyses were performed using GraphPad Prism 9.0 and R (v4.2.1). Data distribution was assessed using the Shapiro–Wilk test. For parametric data, Student’s *t*-test (two-tailed) or Welch’s *t*-test (for unequal variances) were used for pairwise comparisons. Non-parametric data were analyzed using the Mann–Whitney *U* test. Multiple group comparisons were corrected using Dunnett’s or Tukey’s post-hoc tests following one-way or two-way ANOVA. A *p*-value of less than 0.05 was considered statistically significant.

## Supplementary information

Supplementary Figure Legend

The original western blots

SUPPLEMENTAL MATERIAL

Supplemental Figure 1

Supplemental Figure 2

Supplemental Figure 3

Supplemental Figure 4

Supplemental Figure 5

## Data Availability

All data supporting the findings of this study are available within the article and its supplementary information files.
