# Supplementary material for: SOX4 reprograms fatty acid metabolism through the CHREBP to inhibit ferroptosis in hepatocellular carcinoma
Source: Cell Death Discov. 2025 May 21;11:246. doi: 10.1038/s41420-025-02527-4 (PMC12095664; doi:10.1038/s41420-025-02527-4)
Supplement: Supplementary file 1 — Supplementary Figure Legend [file 41420_2025_2527_MOESM1_ESM.docx]

**Supplementary Figure Legend**

**Figure S1** (A-B) Efficiency of SOX4 knockdown in HepG2 cells confirmed by RT-qPCR and Western blotting. (C-D) Validation of stable SOX4 overexpression in Huh7 cells through RT-qPCR and Western blotting. **P < 0.01, ***P < 0.001.

**Figure S2** (A) Heatmap showing significantly differential gene expression following SOX4 knockdown in HCC cells. (B) PCA plot demonstrating clear separation between the NC group and the shSOX4 group used for NGS analysis. (C) PPI network illustrating the potential protein-protein interactions among selected differentially expressed genes.

**Figure S3** (A) The CCK - 8 assay was used to compare the proliferation of SOX4 - knockdown HepG2 cells with or without exogenous C18:1 (cis - 9) supplementation.（B） Flow cytometry was used to evaluate the ROS level in SOX4 - knockdown HepG2 cells treated with C18:1 (cis - 9).

**Figure S4** (A) RNA sequencing data showing that SOX4 knockdown significantly reduces MLXIPL (ChREBP) expression. (B) SOX4 knockdown alters the expression of fatty acid metabolism-related proteins ACLY, SCD, and FASN. (C) The specific SCD1 inhibitor GSK1940029 reduces the proliferative effects of SOX4 overexpression, as demonstrated by the CCK-8 assay.**P < 0.01, ***P < 0.001.

**Figure S5** (A-B) Efficiency of ChREBP knockdown in HepG2 cells confirmed by RT-qPCR and Western blotting. (C-D) Validation of stable ChREBP overexpression in HepG2 cells through RT-qPCR and Western blotting. **P < 0.01, ***P < 0.001.
