# Supplementary figures and images for: SOX4 reprograms fatty acid metabolism through the CHREBP to inhibit ferroptosis in hepatocellular carcinoma
Source: Cell Death Discov. 2025 May 21;11:246. doi: 10.1038/s41420-025-02527-4 (PMC12095664; doi:10.1038/s41420-025-02527-4)

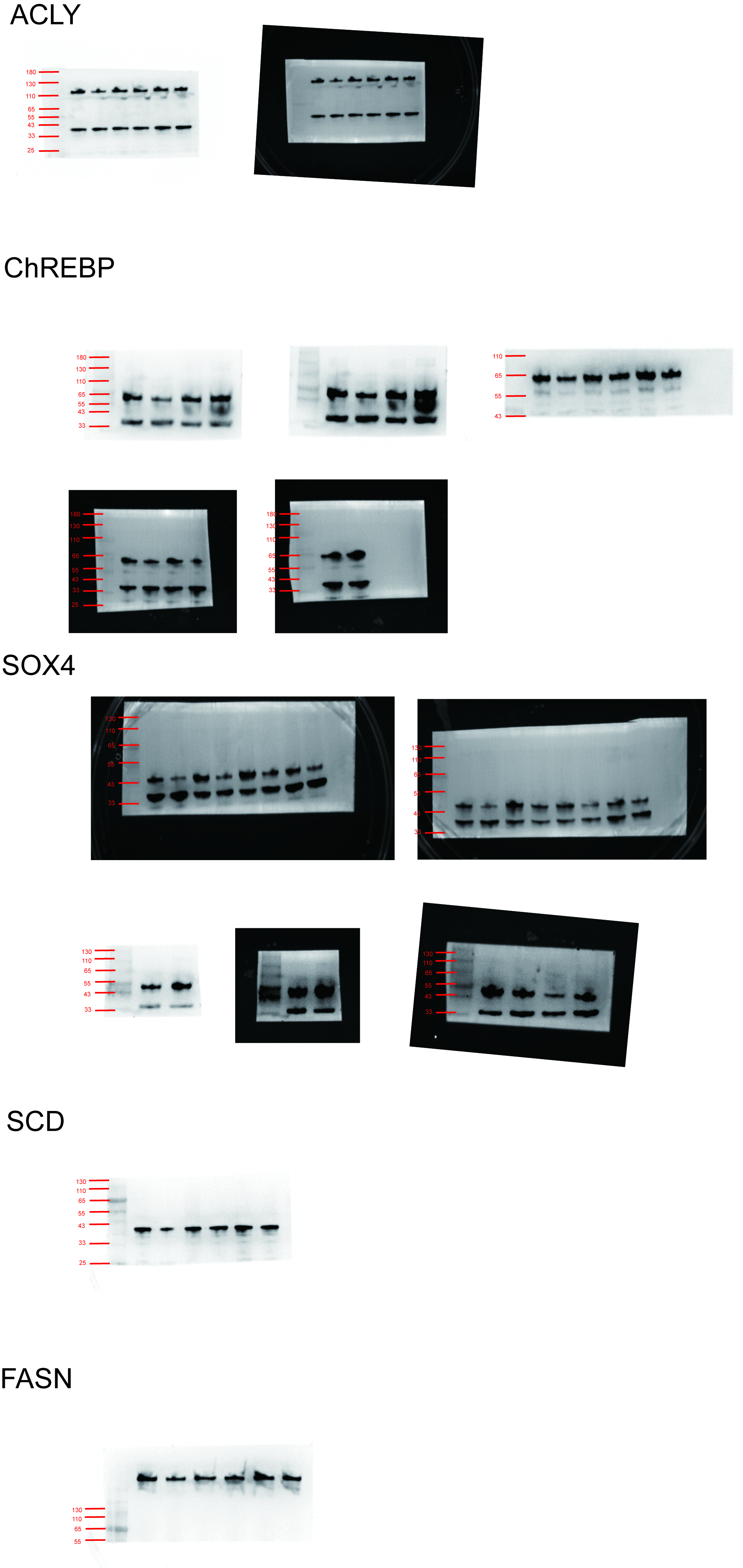

Supplement: Supplementary file 2 — The original western blots [file 41420_2025_2527_MOESM2_ESM.tif]

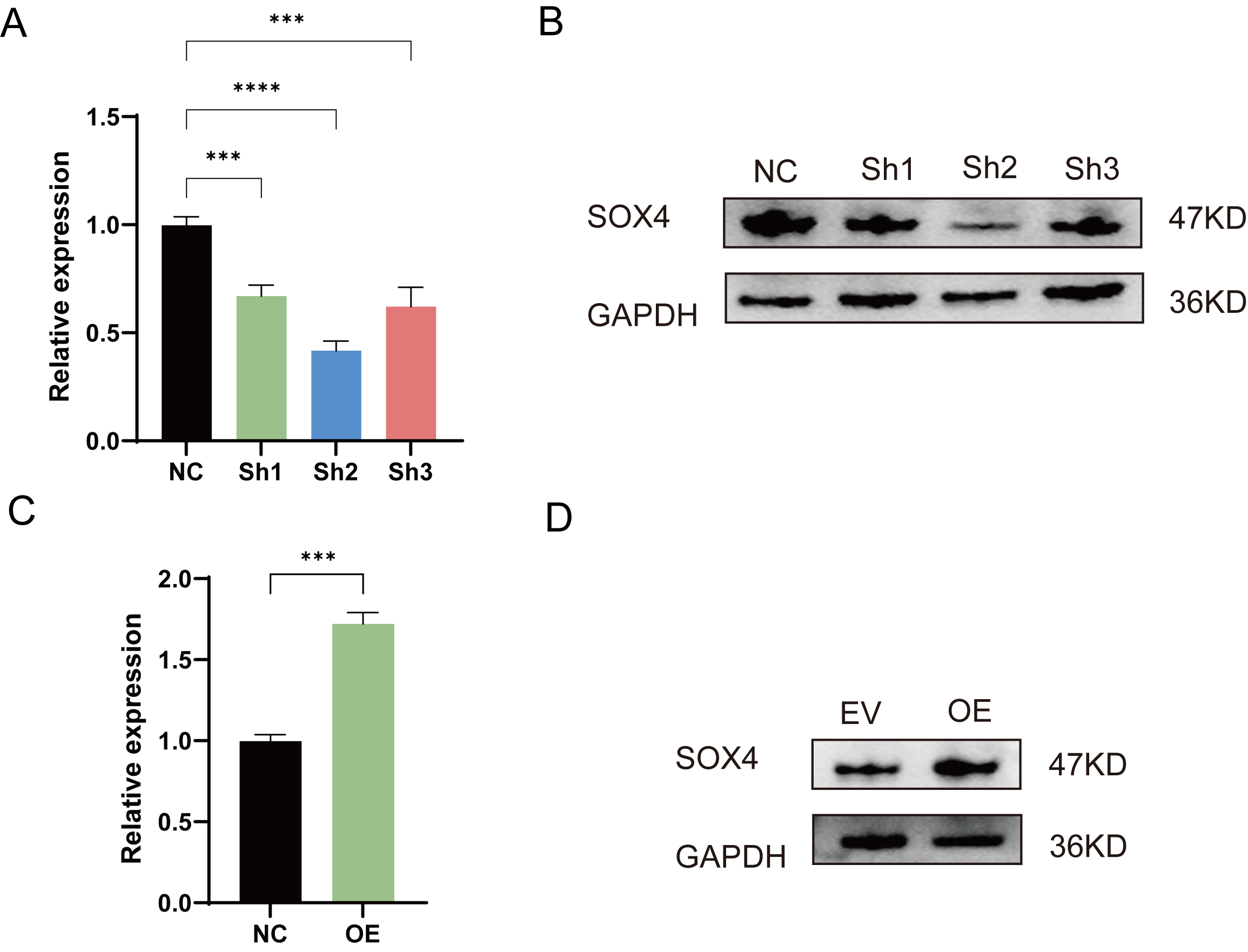

Supplement: Supplementary file 4 — Supplemental Figure 1 [file 41420_2025_2527_MOESM4_ESM.tif]

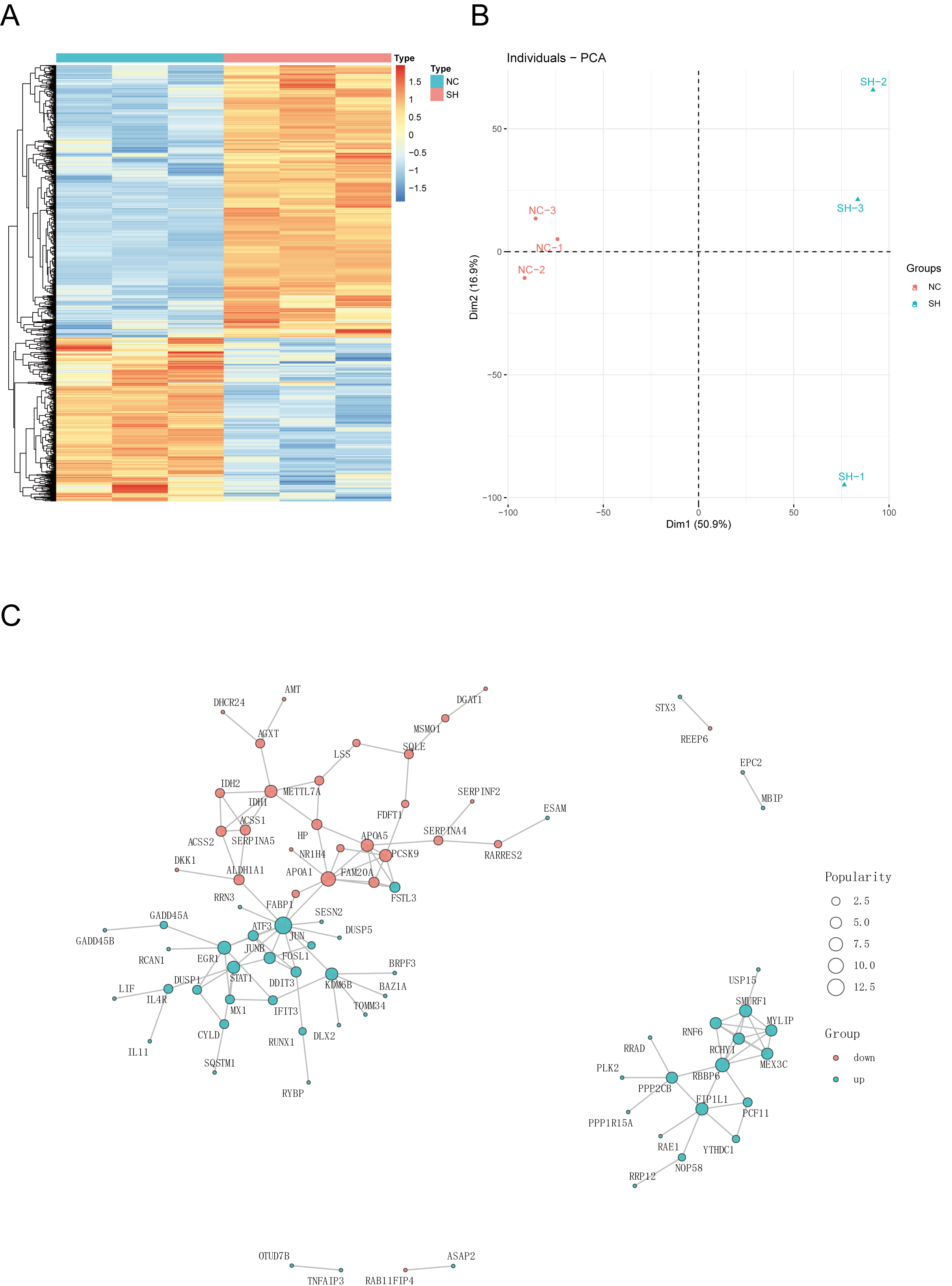

Supplement: Supplementary file 5 — Supplemental Figure 2 [file 41420_2025_2527_MOESM5_ESM.tif]

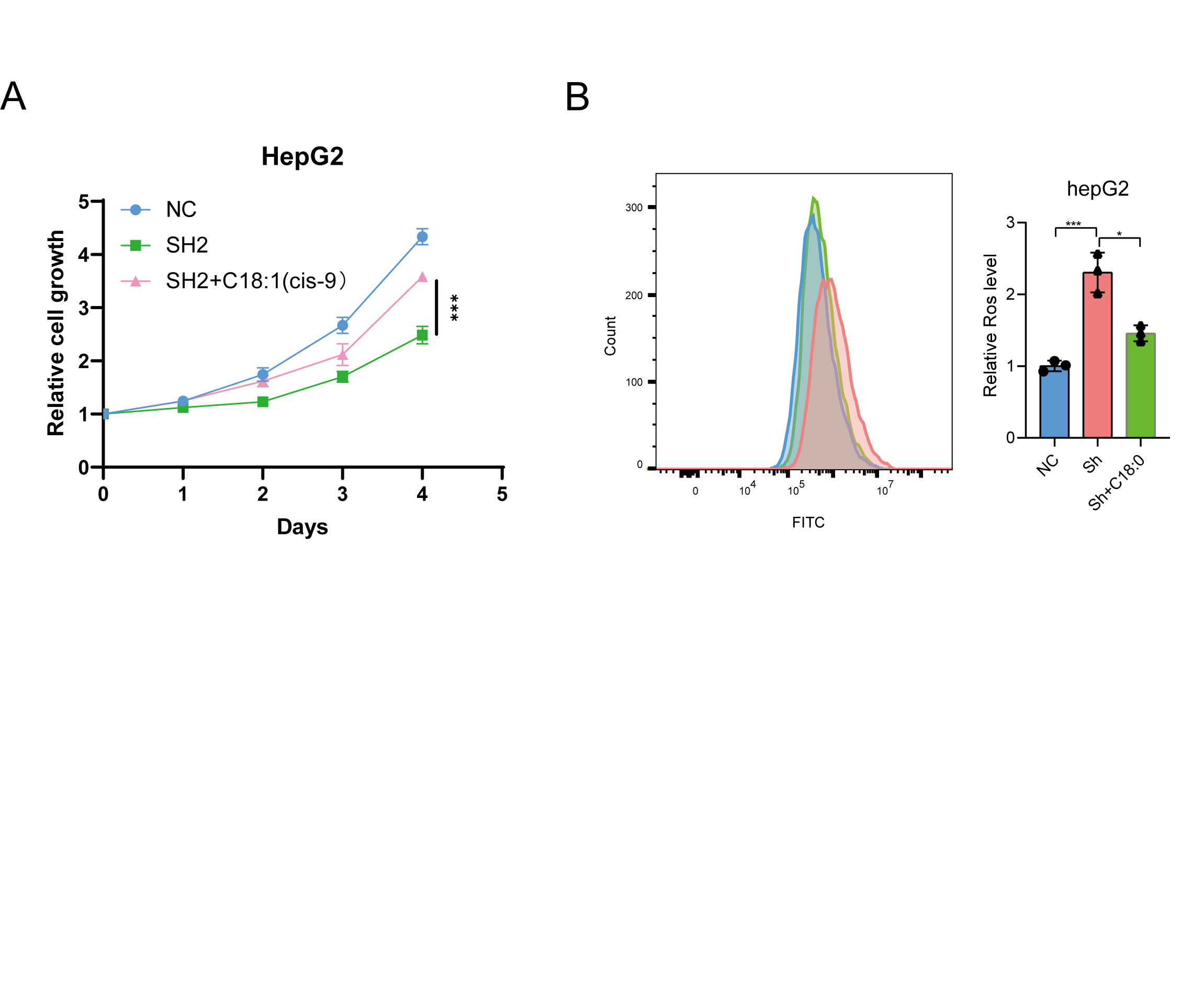

Supplement: Supplementary file 6 — Supplemental Figure 3 [file 41420_2025_2527_MOESM6_ESM.tif]

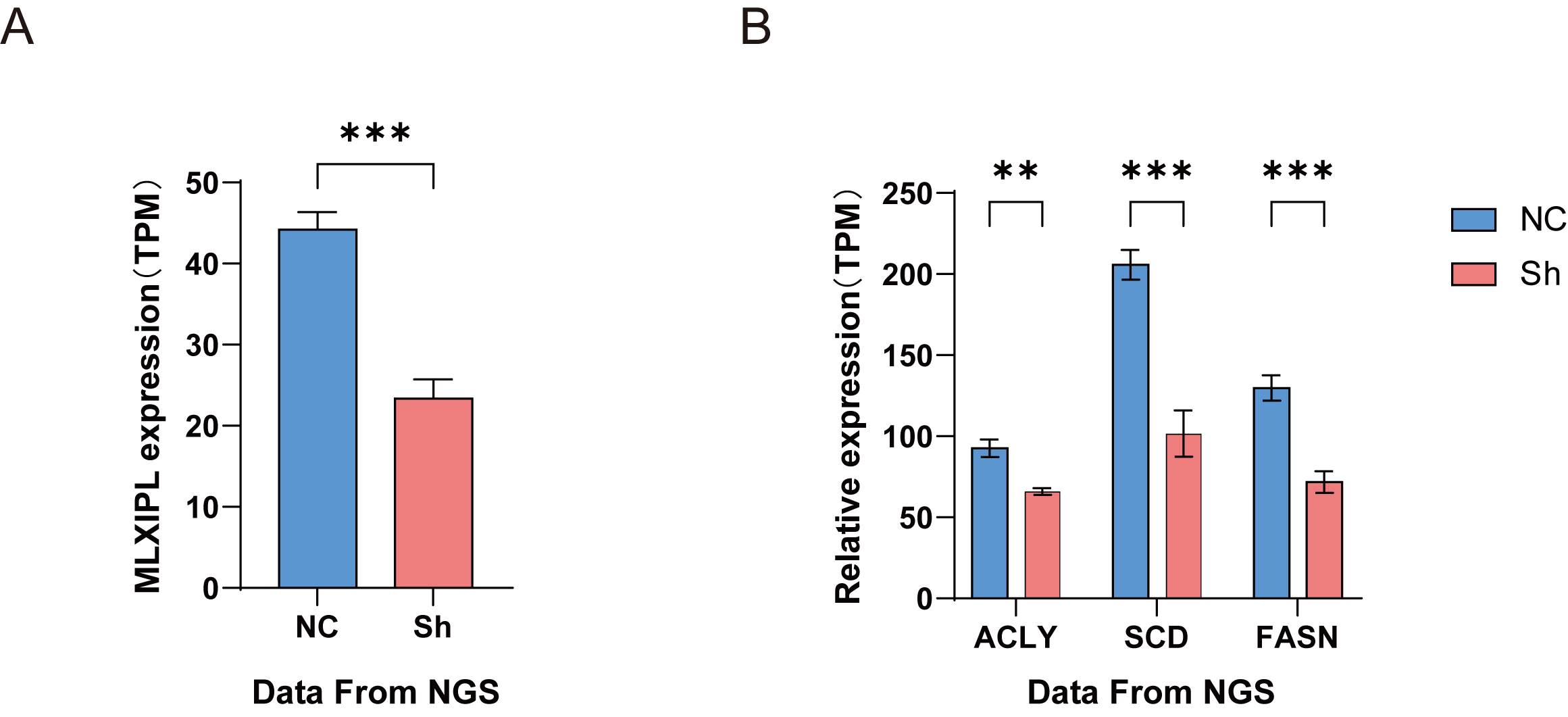

Supplement: Supplementary file 7 — Supplemental Figure 4 [file 41420_2025_2527_MOESM7_ESM.tif]

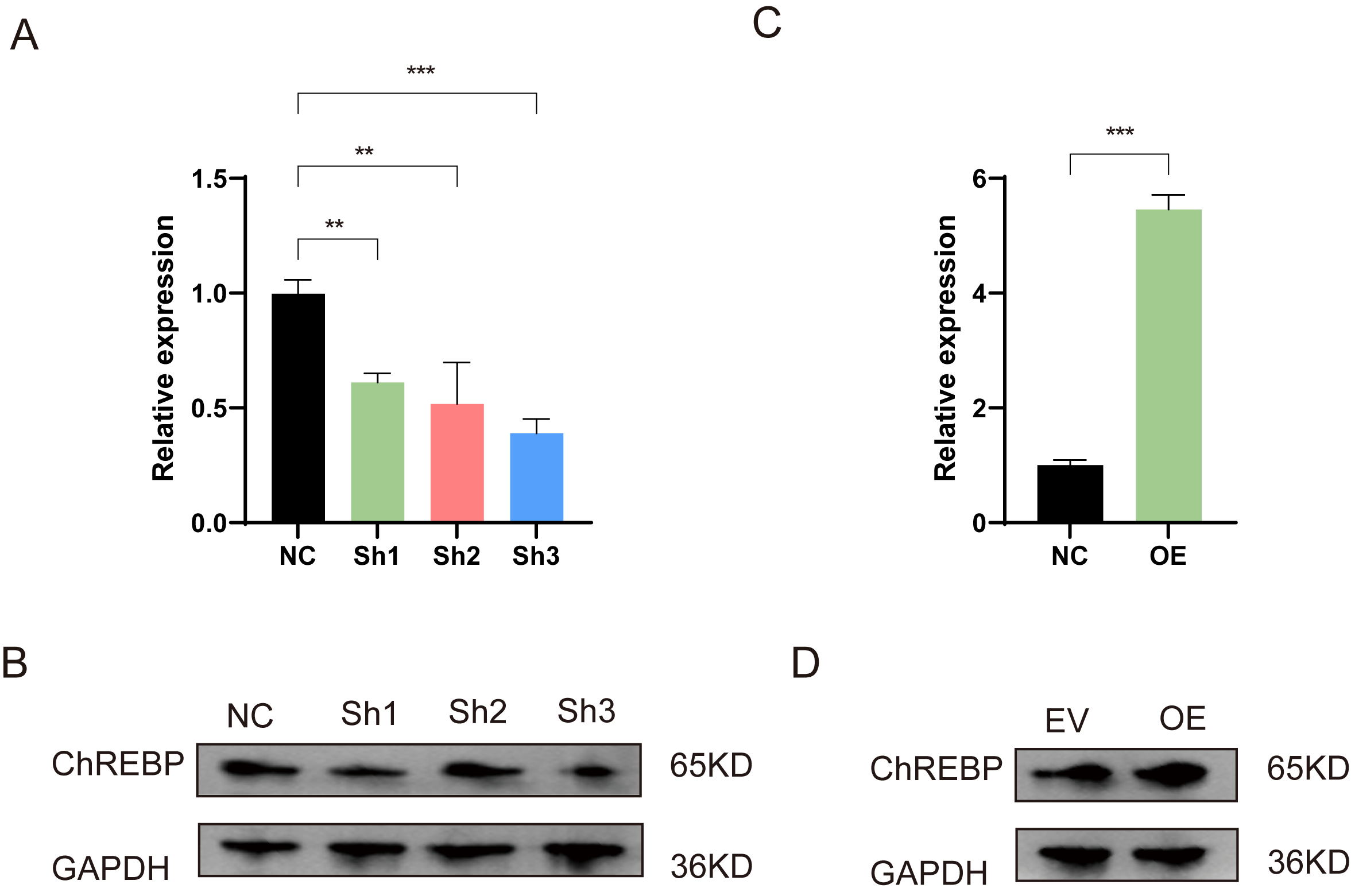

Supplement: Supplementary file 8 — Supplemental Figure 5 [file 41420_2025_2527_MOESM8_ESM.tif]
