# Supplementary material for: SOX4 reprograms fatty acid metabolism through the CHREBP to inhibit ferroptosis in hepatocellular carcinoma
Source: Cell Death Discov. 2025 May 21;11:246. doi: 10.1038/s41420-025-02527-4 (PMC12095664; doi:10.1038/s41420-025-02527-4)
Supplement: Supplementary file 3 — SUPPLEMENTAL MATERIAL [file 41420_2025_2527_MOESM3_ESM.docx]

**Supplementary Table 1: qRT-PCR primer sequences in this study**

| **Gene Symbol** | **primerF** | **primerR** |
| --- | --- | --- |
| SOX4 | AGCGACAAGATCCCTTTCATTC | CGTTGCCGGACTTCACCTT |
| ChREBP | AAGATCCGCCTGAACAACG | CACTTGTGGTATTCCCGCATC |
| SCD | GCCCCTCTACTTGGAAGACGA | AAGTGATCCCATACAGGGCTC |
| FASN | AAGGACCTGTCTAGGTTTGATGC | TGGCTTCATAGGTGACTTCCA |
| ACLY | ATCGGTTCAAGTATGCTCGGG | GACCAAGTTTTCCACGACGTT |

**Supplementary Table 2：Primary and secondary antibodies used in this study**

| **Antibody** | **Host** | **Cat** | **Company** |
| --- | --- | --- | --- |
| Anti-SOX4 | Rabbit | 27414-1-AP | Proteintech |
| Anti-ChREBP | Rabbit | 13256-1-AP | Proteintech |
| Anti-SCD | Rabbit | P05251 | promab |
| Anti-FASN | Rabbit | P12493 | promab |
| Anti-ACLY | Rabbit | 15421-1-AP | Proteintech |
| Anti-GPADH | Rabbit | 10494-1-AP | Proteintech |

**Supplementary Table 3 Drugs and reagents**

| Drug / Reagent | Source | Identifier / formulation |
| --- | --- | --- |
| Erastin | Targetmol | Catalog No: 571203-78-6 |
| Nile Red | MedChemExpress | Catalog No: HY-D0718 |
| Ferrostatin-1 | Targetmol | Catalog No: 347174-05-4 |
| GPX4-IN-3 | MedChemExpress | Catalog No: HY-141809 |

**Supplementary Table 3 Correlation Between HALLMARKs and SOX4 in HCC**

| **Hallmarks** | **Correlation** | **-(log10P)** |
| --- | --- | --- |
| WNT_BETA_CATENIN_SIGNALING | 0.420771065271371 | 18.76721508 |
| ANGIOGENESIS | 0.380384478342789 | 15.21591369 |
| APICAL_JUNCTION | 0.368562532326767 | 14.26561855 |
| NOTCH_SIGNALING | 0.354673165356547 | 13.1979646 |
| HEDGEHOG_SIGNALING | 0.350651235466222 | 12.89842321 |
| EPITHELIAL_MESENCHYMAL_TRANSITION | 0.331613648608644 | 11.53752439 |
| MITOTIC_SPINDLE | 0.326827893724601 | 11.20990384 |
| APICAL_SURFACE | 0.30953685078723 | 10.07328356 |
| TGF_BETA_SIGNALING | 0.296725729450131 | 9.277566312 |
| G2M_CHECKPOINT | 0.294789766764291 | 9.160682748 |
| E2F_TARGETS | 0.219074179953655 | 5.243441621 |
| ALLOGRAFT_REJECTION | 0.193367485736633 | 4.186433175 |
| IL2_STAT5_SIGNALING | 0.180247489517764 | 3.696996275 |
| MYOGENESIS | 0.177440756006818 | 3.596587172 |
| INFLAMMATORY_RESPONSE | 0.146628980742593 | 2.591458025 |
